# The Effectiveness of an Interactive 3-Dimensional Computer Graphics Model for Medical Education

**DOI:** 10.2196/ijmr.2172

**Published:** 2012-07-09

**Authors:** Bayanmunkh Battulga, Takeshi Konishi, Yoko Tamura, Hiroki Moriguchi

**Affiliations:** 1 Department of Medical InformaticsInstitute of Health BiosciencesUniversity of Tokushima graduate schoolTokushimaJapan

**Keywords:** Medical education, electronic health information, interactive 3D CG, educational effectiveness, Web-based learning management system, satellite network

## Abstract

**Background:**

Medical students often have difficulty achieving a conceptual understanding of 3-dimensional (3D) anatomy, such as bone alignment, muscles, and complex movements, from 2-dimensional (2D) images. To this end, animated and interactive 3-dimensional computer graphics (3DCG) can provide better visual information to users. In medical fields, research on the advantages of 3DCG in medical education is relatively new.

**Objective:**

To determine the educational effectiveness of interactive 3DCG.

**Methods:**

We divided 100 participants (27 men, mean (SD) age 17.9 (0.6) years, and 73 women, mean (SD) age 18.1 (1.1) years) from the Health Sciences University of Mongolia (HSUM) into 3DCG (n = 50) and textbook-only (control) (n = 50) groups. The control group used a textbook and 2D images, while the 3DCG group was trained to use the interactive 3DCG shoulder model in addition to a textbook. We conducted a questionnaire survey via an encrypted satellite network between HSUM and Tokushima University. The questionnaire was scored on a 5-point Likert scale from strongly disagree (score 1) to strongly agree (score 5).

**Results:**

Interactive 3DCG was effective in undergraduate medical education. Specifically, there was a significant difference in mean (SD) scores between the 3DCG and control groups in their response to questionnaire items regarding content (4.26 (0.69) vs 3.85 (0.68), *P *= .001) and teaching methods (4.33 (0.65) vs 3.74 (0.79), *P *< .001), but no significant difference in the Web category. Participants also provided meaningful comments on the advantages of interactive 3DCG.

**Conclusions:**

Interactive 3DCG materials have positive effects on medical education when properly integrated into conventional education. In particular, our results suggest that interactive 3DCG is more efficient than textbooks alone in medical education and can motivate students to understand complex anatomical structures.

## Introduction

The Internet has become a social platform where millions of health consumers access and share health information [1]. One such medium, eHealth [2], has brought about improvements in public health and the health care system. Medical information technology has influenced the medical profession during the last decade and will continue to make advances. For example, 3-dimensional (3D) presentation of information is being increasingly used in medical education and health care [3].

Modern human anatomy pedagogy includes cadaver dissection, multimedia presentations, practical procedures, surface and clinical anatomy, and radiological imaging [4]. Cadaver dissection is the standard method of learning anatomy and allows for a haptic understanding of 3D anatomical structures [5,6] but is expensive and time consuming [7], and curriculum hours for anatomy decrease yearly [8,9]. The current trend in medical education is to achieve anatomical understanding in less time by using information and communication technology. Information and communication technology can approximate human anatomy and motion through 3-dimensional computer graphics models (3DCG), but achieving realistic anatomy and motion is more difficult.

Students often have difficulty achieving a spatial understanding of 3D anatomy from 2-dimensional (2D) images and text. This can increase cognitive load and hinder anatomy learning for students with poor spatial skills [10-13]. 3DCG models promise to overcome many of these educational challenges. Mayer’s cognitive theory of multimedia learning states that students learn best by using both images and words in an electronic learning environment [14-16]. 3DCG visually provides semireal information to users, thus enabling them to understand the content easily, and the interactivity of 3DCG content improves comprehension. 3DCG animation and interactive 3DCG have been developed at several institutions [17,18]. Kobayashi et al reported that it was easier and more accurate to explain details of surgery using 3DCG animation than 2D illustrations [19]. In addition, methods have been developed that enable users to make highly specialized 3DCG content on the Web [20].

This study was conducted by researchers at the Health Sciences University of Mongolia (HSUM) and the University of Tokushima, Japan. We chose a high-speed satellite communication network because the Internet has not completely spread to rural areas of Mongolia, which is the fifth-largest country in Asia with 2.6 million people (as of 2007). Mongolia has clear skies and annual precipitation as low as 200 mm per year, creating ideal conditions for satellite communication. Our study was selected by the Ministry of Internal Affairs and Communications of Japan as an experimental application for data collection and as a developmental application for satellite communication authorized by the Association of Radio Industries and Businesses for Japan [21].

Improvements in personal computer performance have led to an increase in the development of 3DCG content. The use of 3DCG models has advantages over traditional anatomy instruction methods; however, their development and adoption are time consuming and costly. Thus, new educational information and communication technology instruction methods are needed. To this end, we aimed to determine the educational effectiveness of interactive 3DCG using an interactive 3DCG shoulder model.

## Methods

### Development of the Interactive 3DCG Model

We selected the shoulder for this experiment given its anatomical complexity and because it is considered one of the most difficult joints for medical students to understand in human anatomy. The Department of Anatomy and Developmental Neurobiology, University of Tokushima carefully examined anatomical accuracy, such as the relative spatial relationship of each structure, at every development stage to ensure that our interactive 3DCG models would be of high quality and accurate (see Multimedia Appendix 1).

The process of model creation is not trivial. We built the models in LightWave 3D (NewTek, Inc., San Antonio, TX, USA) and exported them as object files to Blender (an open source 3D program; blender.org, Amsterdam, the Netherlands). Blender was used to generate clean U and V space texture maps for the models. The models were then sent back to LightWave as object files. We created the textures in Photoshop (Adobe Systems Incorporated, San Jose, CA, USA) from photo references, applied them to U and V space maps in LightWave 3D, then exported them in Filmbox format and imported them into Unity3D [22]. Shoulder movements were added in LightWave and exported as Filmbox animation data. Once the model and animation data were imported into Unity3D, we wrote scripts to allow interaction with the models. Unity’s workflow allows swapping of models if changes have been made in LightWave 3D. The initial animation of shoulder movement was quite rigid, so we used a motion capture system to achieve more natural motion.

### Motion Capture System

We used the Vicon MX motion capture system (Vicon Motion Systems, Oxford, United Kingdom) at Tokushima University Hospital [23]. Motion capture data were collected at 150 Hz using a near-infrared (780 nm) passive 8-camera system (Vicon MX T20; Vicon Motion Systems). A 3D position sensor captured the reflected rays of 9.5 mm diameter reflective markers. Nexus 1.4.1 (Vicon Motion Systems) software recorded 3D positions of the markers and extracted vector data. We took screenshots of the motion capture vector data and then moved the shoulder to match those screenshots for each part of the motion. We correlated the skeleton to the visual data, taking screenshots of data over time. This process improved the smoothness of shoulder movements.

### 3DCG Model User Interface and Textbook

**Figure 1:**
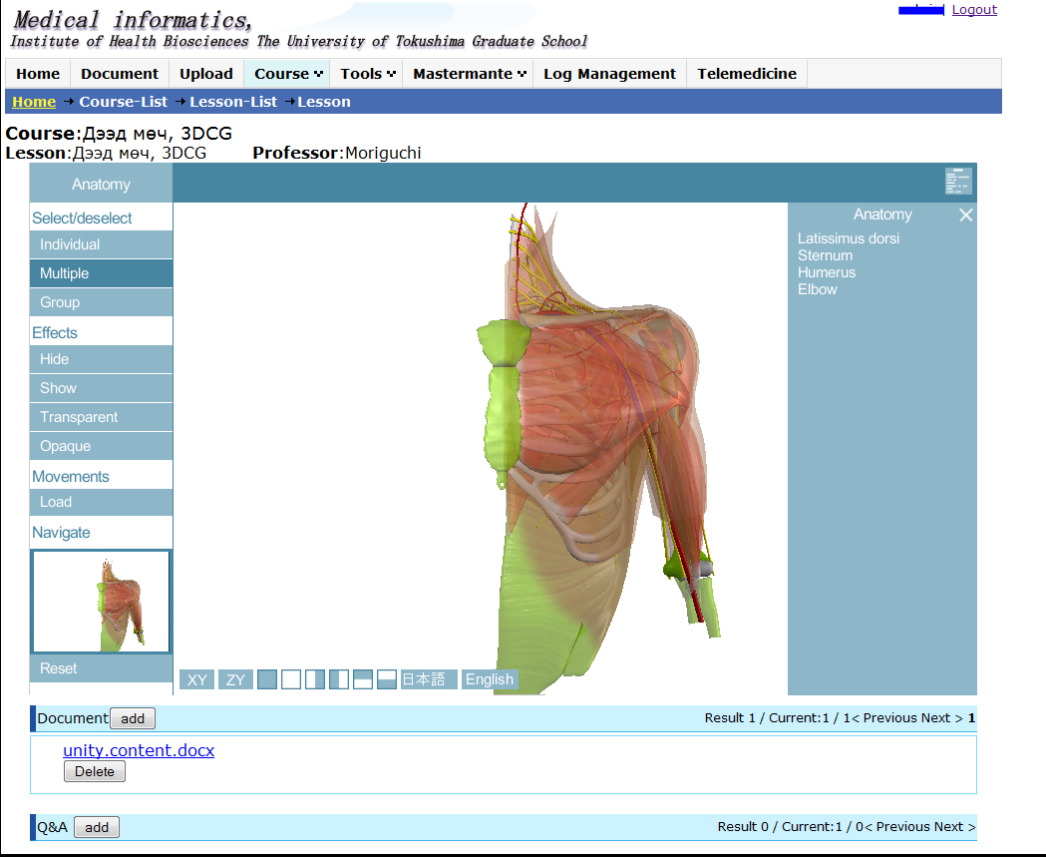
Anatomical view of the 3-dimensional computer graphic showing the shoulder area.

**Figure 2:**
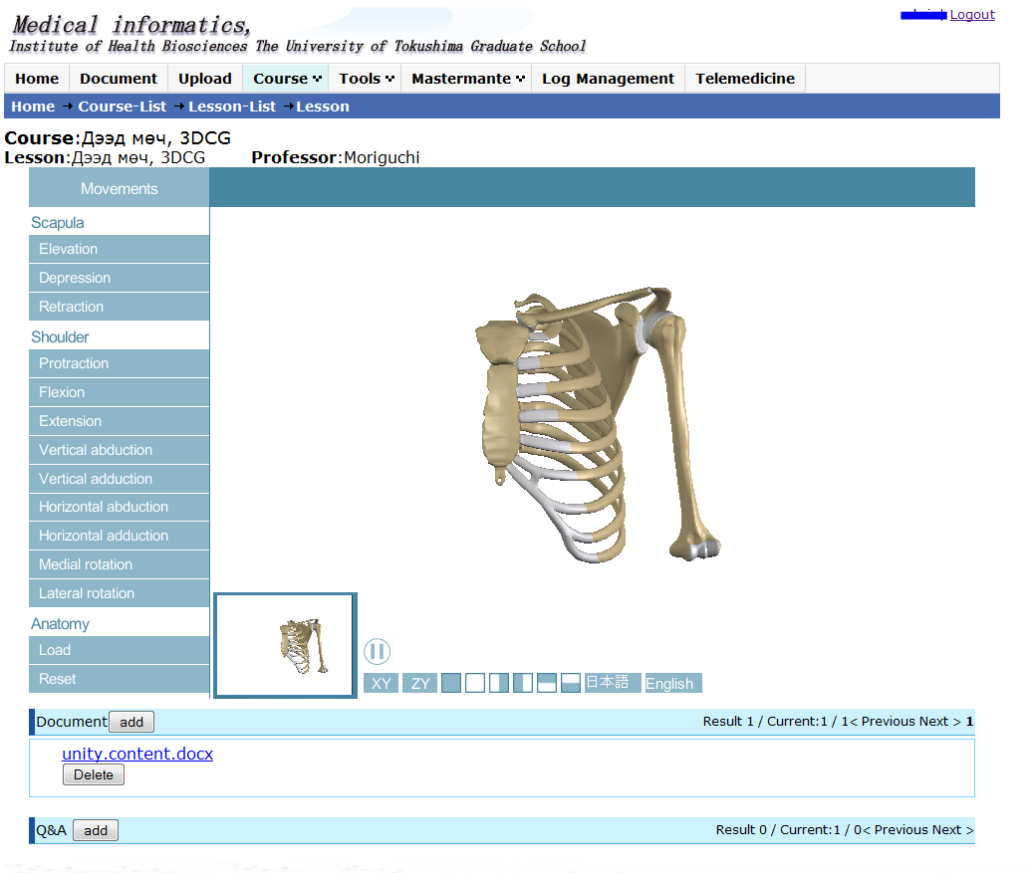
Movement view of the 3-dimensional computer graphic of the shoulder bones and upper extremity.

The menu for the 3DCG interactive manipulation tools on the left side of the screen has two functional components: one for anatomy and the other for shoulder movements, with labeling ability in English or Japanese. Figure 1 shows the 3DCG of the area surrounding the shoulder and the terminology of each part on the upper right corner. Figure 2 shows the movements of the shoulder bones and upper extremity. Several important movements are available, including elevation, depression, retraction, protraction, flexion, extension, vertical abduction, vertical adduction, horizontal abduction, and horizontal adduction. Both views allow the user to zoom in and out, and to focus on a region of interest. Users are also able to quickly rotate or move to a specific angle such as anterior, lateral, and posterior. To study specific anatomical regions, the tool enables users to select single or multiple objects and hide them or make them semitransparent. We also developed an original textbook for this study. To provide a suitable condition for comparison, we replaced the black and white figures traditionally used in lectures at HSUM, which were drawn from a standard anatomical viewpoint, with a new textbook based on 3DCG images selected from the interactive 3DCG system. We reproduced 34 images (anterior, lateral, posterior, and other views with appropriate angle and magnitude for evaluation) from the 3DCG shoulder models in gif format for the textbook and added appropriate text to explain the images.

**Figure 3:**
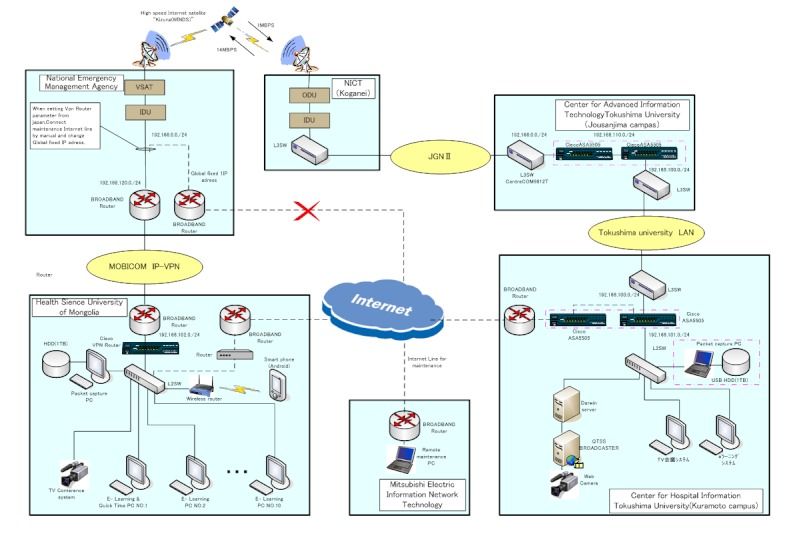
Network architecture.

We used Wideband InterNetworking engineering test and Demonstration Satellite for the communication system, which was jointly developed by the Japan Aerospace Exploration Agency and the National Institute of Information and Communications Technology of Japan. A small antenna 1.2 m in diameter receives up to 155 Mbps of data and transmits up to 6 Mbps, while an antenna approximately 5 m in diameter enables 2-way communication up to 1.2 Gbps [24]. For our study, the Japan Aerospace Exploration Agency allocated reception bands from 1.31 Mbps to 1.38 Mbps for the uplink connection, and from 15.0 Mbps to 20.7 Mbps for the downlink connection. The link was encrypted using an Internet Protocol Security virtual private network based on a Cisco ASA 5505 router (Cisco Systems Inc., San Jose, CA, USA) provided by a joint research project with Mitsubishi Electric Information Network Corporation (Figure 3).

### Throughput of the Satellite Network

Round-trip time is the signal delay between the University of Tokushima and HSUM via the satellite network and the Internet. The round-trip time was between 1499.3 ms and 643.8 ms, with an average of 729.6 ms. The congestion window is a Transmission Control Protocol parameter that regulates the send window. The congestion window of Windows XP ranges from 16 KB in default to 64 KB. Therefore, the maximum throughput available in the Transmission Control Protocol is 0.795 Mbps, which is used for Hypertext Transfer Protocol. However, the actual throughput value was 0.384 Mbps or less.

### Web-Based Learning Management System

All activity associated with the course was hosted on a Linux-based server running the Apache Web server (Apache Software Foundation, Los Angeles, CA, USA), the PostgreSQL database server (PostgreSQL Global Development Group, http://www.postgresql.org/), and the CentOS operating system (CentOS Project, http://www.centos.org/). Our proprietary learning management system (LMS) allowed us to create a course website with a unique log-in password and ID for each student. We developed the interactive 3DCG model, textbook, and questionnaires, and embedded them into this LMS. These components were used for the related experimental section. We applied Java Web applications for the system and adopted Unity3D for the 3DCG container, which is an integrated 3D platform for 3D games and interactive content on the Web.

### Study Design

**Figure 4:**
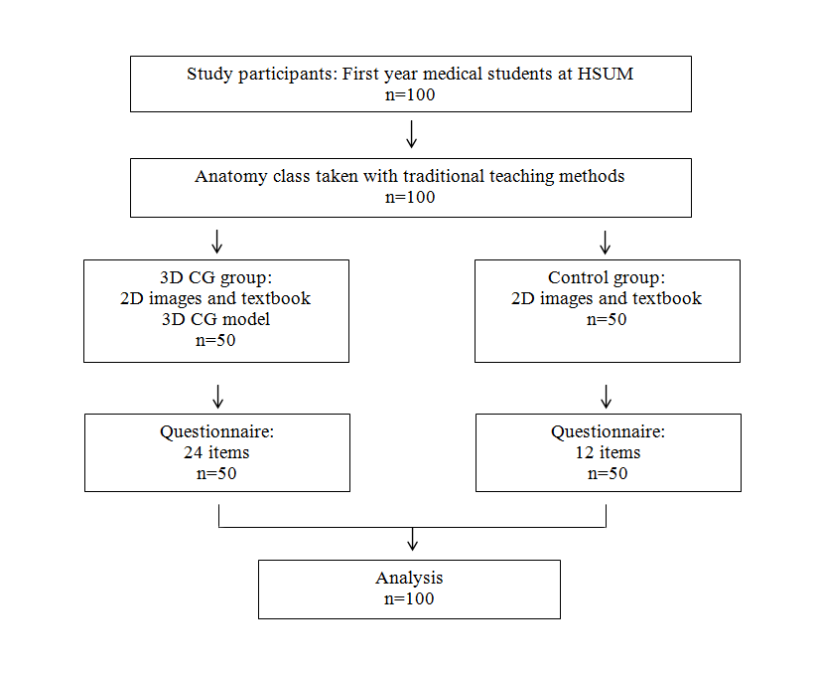
Study design. 2D = 2-dimensional, 3DCG = 3-dimensional computer graphics, HSUM = Health Sciences University of Mongolia.

Figure 4 shows the study design. All participants who had taken anatomy classes and finished cadaver dissection 3 months previously received a brief introduction to the LMS before the experiment. The study was conducted over a 1-week period. We divided participants into a 3DCG group and a textbook-only (control) group. The control and 3DCG groups were also given instructions on how to use the textbook, and the 3DCG group was given additional training to manipulate the interactive 3DCG model. Each participant received a 1-hour training session. Finally, all participants completed a questionnaire on the LMS.

### Statistical Analysis

Statistical analysis was performed using SPSS (version 16.0 for Windows; IBM Japan Inc., Tokyo, Japan). We conducted both the independent-samples *t *test and the Mann-Whitney *U *test to compare the two groups, with *P *< .05 defined as statistically significant. The internal consistency coefficient (Cronbach alpha) was calculated for both groups.

## Results

### Participants

A total of 100 first-year medical students (27 men and 73 women; Table 1) from HSUM volunteered, gave informed consent to participate in the study, and were randomly assigned to either the 3DCG group (n = 50) or the control group (n = 50). The mean age of participants was 18.1 (SD 1.1) years (men: 17.9 (SD 0.6) years, women: 18.1 (SD 1.1); range 16–25 years). Participants were freshmen at HSUM and were instructed in practical computer skills for half a semester (64 hours).

**Table 1:** Gender distribution in the two study groups (n = 100).

Gender	3DCG^a ^group (n = 50)	Control group (n = 50)
Male	13	14
Female	37	36

^a ^3-dimensional computer graphics.

### e-Learning Questionnaire

The questionnaire had 24 items grouped into four categories: content (3 items), teaching methods (6 items), Web (3 items), and 3DCG model interface (12 items). Each item was scored based on a 5-point Likert scale: strongly agree (score 5), agree (score 4), neutral (score 3), disagree (score 2), and strongly disagree (score 1).

**Table 2:** Questionnaire scores for content, teaching methods, and Web items (n = 100).

Questionnaire item		Response					Mean score
		Strongly agree	Agree	Neutral	Disagree	Strongly disagree	
Q1	The content is useful.	30	51	19	0	0	4.11
Q2	The content is easy to read and understand.	19	55	22	4	0	3.89
Q3	The content is well formatted and well designed.	25	52	21	2	0	4.00
Q4	The support for my study is effective.	31	52	17	0	0	4.14
Q5	This teaching method can improve my knowledge.	27	59	13	1	0	4.12
Q6	This teaching method can help my learning.	27	53	19	1	0	4.06
Q7	This teaching method motivates me when I learn.	38	40	18	4	0	4.12
Q8	This teaching method gives me enough time in the lesson.	24	40	26	9	1	3.77
Q9	This lesson is appropriate for my learning demand.	31	43	25	1	0	4.04
Q10	The webpage is attractive.	25	58	15	1	1	4.05
Q11	The screen design is clear.	26	53	18	2	1	4.01
Q12	The menu is easy to use.	25	57	17	0	1	4.05

Table 2 shows that mean scores ranged from 3.77 to 4.14. For each item, 19%–38% of participants responded strongly agree, 40%–59% responded agree, 13%–26% responded neutral, 0%–9% responded disagree, and 0%–1% responded strongly disagree. As a result, 64%–86% of participants responded either strongly agree or agree to each item.

**Table 3:** Questionnaire scores for the 3-dimensional computer graphics (3DCG) model interface (n = 50).

Questionnaire item		Response					Mean score
		Strongly agree	Agree	Neutral	Disagree	Strongly disagree	
Q13	The interface for interacting with the 3D^a ^content is accessible.	17	26	7	0	0	4.2
Q14	The volume of information in the 3D module is appropriate.	15	26	8	1	0	4.1
Q15	I am satisfied with the 360° rotation of the model.	32	17	1	0	0	4.62
Q16	I am satisfied with the selection menu.	13	35	2	0	0	4.22
Q17	I am satisfied with the show-and-hide function.	21	20	9	0	0	4.24
Q17	I am satisfied with the transparent and opaque function.	13	30	7	0	0	4.12
Q19	I am satisfied with the zoom function.	29	18	3	0	0	4.52
Q20	The screen size is appropriate.	11	26	13	0	0	3.96
Q21	I am satisfied with clicking the mouse to show anatomical terminology.	29	20	1	0	0	4.56
Q22	I am interested in 3DCG.	33	14	3	0	0	4.6
Q23	I am satisfied with the movement menu.	26	21	3	0	0	4.46
Q24	The 3DCG content is of high quality.	18	31	1	0	0	4.34

^a ^3-dimensional.

Table 3 shows that mean scores for the 3DCG module range from 3.96 to 4.62. For each item, 22%–66% responded strongly agree, 28%–70% responded agree, 2%–26% responded neutral, and 0%–1% responded disagree. None of the participants responded strongly disagree to the statements. Overall, 74%–98% of the participants responded either strongly agree or agree. The reliability of the entire questionnaire was acceptable (Cronbach alpha = .902).

**Table 4:** Comparison of mean (SD) questionnaire scores^a ^between 3-dimensional computer graphics (3D G) and control groups.

Category	Questionnaire item	3DCG group	Control group	*P *value
Content	The content is useful.	4.34 (0.63)	3.88 (0.69)	.001^b^
	The content is easy to read and understand.	3.90 (0.70)	3.88 (0.80)	.97
	The content is well formatted and well designed.	4.18 (0.75)	3.82 (0.69)	.01^b^
Teaching methods	The support for my study is effective.	4.36 (0.63)	3.92 (0.66)	.001^b^
	This teaching method can improve my knowledge.	4.40 (0.61)	3.84 (0.58)	<.001^b^
	This teaching method can help my learning.	4.28 (0.64)	3.84 (0.71)	.002^b^
	This teaching method motivates me when I learn.	4.52 (0.58)	3.72 (0.88)	<.001^b^
	This teaching method gives me enough time in the lesson.	4.14 (0.70)	3.40 (1.03)	<.001^b^
	This lesson is appropriate for my learning demand.	4.32 (0.71)	3.76 (0.74)	<.001^b^
Web	The webpage is attractive.	4.26 (0.56)	3.84 (0.82)	.006^b^
	The screen design is clear.	4.10 (0.61)	3.92 (0.92)	.45
	The menu is easy to use.	4.14 (0.61)	3.96 (0.81)	.29

^a ^5-point Likert scale from strongly disagree (score 1) to strongly agree (score 5).

^b ^Significant difference (Mann-Whitney *U *test).

**Figure 5:**
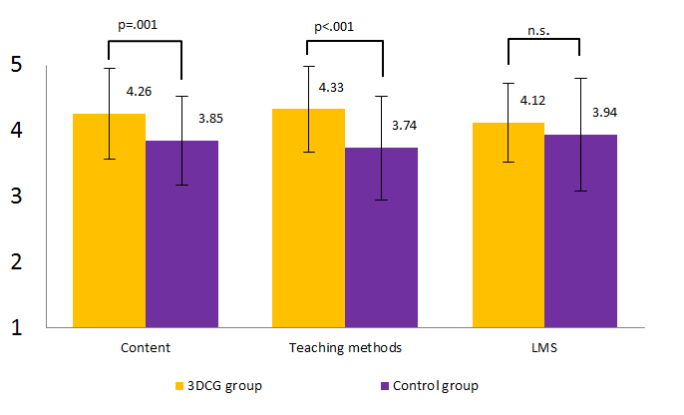
Mean scores of the 3-dimensional computer graphics (3DCG) and control groups for the three main categories. LMS = learning management system, n.s. = not significant.

Table 4 and Figure 5 present the mean (SD) scores. Table 4 compares scores between the 3DCG and control groups for each item. The 3DCG group scores ranged from 3.90 (SD 0.70) to 4.52 (SD 0.58), and control group scores ranged from 3.40 (SD 1.03) to 3.96 (SD 0.81). Differences in learning motivation scores were the largest, with the 3DCG group giving an average score 0.8 higher than the control group. Participants’ comments on the advantages of using the interactive 3DCG model included “very interesting,” ”realistic,” “saved time,” “more understandable,” “amazing movements,” and “clarity.” There were also some comments regarding disadvantages, such as the necessity for “muscle movement,” “more detailed anatomy,” and “larger screen size,” which would require compilation to change the frame size.

**Figure 6:**
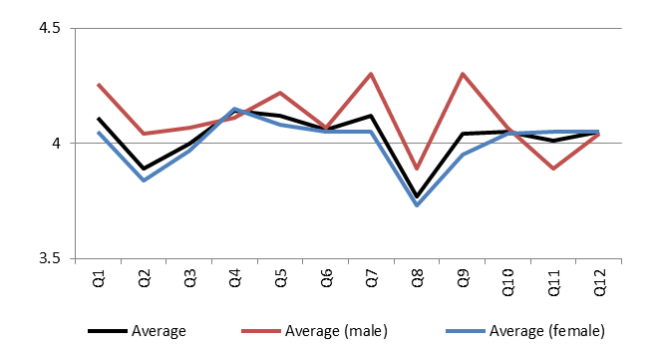
Questionnaire scores by gender.

Combined scores for the three categories are shown in Figure 5. There was a significant difference between the 3DCG and control groups for content (4.26 (SD 0.69) vs 3.85 (SD 0.68); *P *= .001) and teaching methods (4.33 (SD 0.65) vs 3.74 (SD 0.79); *P *< .001). No significant difference was found between the groups in the Web category. The mean score for male participants was higher than that of female participants, except for Q11 (Figure 6). There was no significant difference between men and women for Q1–Q8 and Q10–Q12, but the difference for Q9 was significant.

## Discussion

In this study, we investigated the educational effectiveness of using an interactive 3DCG model as a supplement to conventional learning methods. Our results show that the interactive 3DCG model is effective in undergraduate medical education and can enhance the motivation of medical students. We employed an LMS with two kinds of content. Content for the 3DCG group included Unity3D-based material, while content for the control group included only text and 2D images.

Responses of all participants (n = 100) on common questionnaire items in three categories are presented in Table 2. Items Q1, Q4, Q5, and Q7 had the highest scores, while scores for Q2 and Q8 were relatively low. Strongly agree responses were particularly high for items Q1, Q4, Q7, and Q9. Disagree and strongly disagree comprised less than 10% of all questionnaire item responses. These results indicate that participants generally accepted the LMS, although they may require more time to acclimate to the new learning system.

Table 3 shows that 11 items out of 12 had mean scores higher than 4. The relatively low scores for screen size of the 3DCG may be due to the small size of the Unity framework, which has a fixed size of 950 × 534 pixels. Participants indicated a high interest in the 3DCG model and were satisfied with the 360° rotation, zooming function, ability to show terminology, and movement of the shoulder joint.

Table 4 shows significant differences in 9 out of 12 items. Scores are significantly different for 2 items in the content category. However, there was no difference for Q2, which was expected because textbook images were selected and copied from the 3DCG shoulder models. Q2 also has one of the worst scores in Table 2 and is particularly low compared with other scores in the 3DCG group (Table 4), which could be due to the fact that participants were not accustomed to operating the interactive 3DCG. We believe that participants in the 3DCG group accepted the interactive 3DCG because they found it to be effective and motivating, and it satisfied their learning demands. The significant difference for item Q10 indicates that evaluation of the LMS depends on content quality, such as interactive 3DCG, which attracts interest. The mean score of the 3DCG group (4.26), which is higher than that of the control group (3.84), indicates a strong interest in 3DCG and the necessity of the interactive component, including functions for scaling, changing perspectives, and movement. This may derive from the need to be appropriately positioned to view specific anatomical structures for better comprehension. The 3DCG group gave high scores for items in the teaching methods category, and particularly high scores on items addressing usefulness of content, demonstrating that students desired a better way to view specific anatomical structures. Garg et al [25] investigated the usefulness of computer-mediated anatomical 3D reconstructions in anatomical learning and found that learners with low visuospatial ability performed worse on an anatomical knowledge test following the multiple-view condition than following the key view. They also concluded that the key view is important for understanding 3DCG that has many dominants, which indicate the region of concern of users. On the basis of their key view theory, learners using an interactive 3DCG model that was made for a crucial anatomical site, such as the shoulder, could naturally select any important key views themselves for better understanding of the specific anatomical region. The spontaneous, easy, and unburdened way of searching in key view may promote student learning, while textbooks provide a restricted viewpoint. The significantly different scores in the teaching methods category suggest that participants in the 3DCG group felt the 3DCG model motivated their learning, improved their knowledge, and was effective for studying. Moreover, the model satisfied their learning demands and was helpful for self-study. There were no significant differences in scores for items regarding the design and menu in the Web category because the screens were the same. This might strengthen the validity of the responses provided by the two groups.

Comments from participants also suggested that interactive 3DCG increased the motivation to learn a large number of anatomical structures and clarified anatomy. Students generally dislike memorizing many names and learning the complexity of nerves and blood vessels and how joints move, in a short period of time. Scores of male participants tended to be higher than scores of female participants. Some research has revealed that women have higher computer anxiety [26] and less learning emphasis, strategy [27], computer self-efficacy, perceived usefulness, perceived ease of use, and behavioral intention to use e-learning [28]. Scores on Q9 showed that women had significantly lower learning demands. Men and women should have similar learning attitudes toward 3DCG anatomical content, as shown in these reports. They are also required to acquire terminology that must be remembered, although memorization is difficult in a limited time frame.

Some studies have attempted to evaluate the effectiveness of learning tools. Findings include that animated visual tools are more effective than static visual tools [29], the use of 3D animation leads to better topographical and theoretical understanding [30], 3D multimedia software has a positive impact on dental education [31], and 3D surgical simulators [32] and 3D larynx models [33] can enhance student learning through increased motivation. Our findings are consistent with these reports. Another study [34] showed that 2D visualization was superior to 3D visualization in improving the understanding of organic molecule structures, but scores for the 2D and 3D groups were similar. The report stressed that better results would be achieved under conditions of greater familiarity with 3D. A limitation of our study is that we assessed learning effectiveness using the 3DCG model only by questionnaire. We have not measured the outcome of anatomical understanding.

We conclude that interactive 3DCG materials have positive effects on medical education when properly integrated into conventional education. In particular, the interactive 3DCG motivated participants to understand a complex anatomical structure.

## Multimedia Appendix 1

Examples of screenshots and pictures of the web-based learning management system and development of the interactive 3D CG.

## Abbreviations

2D: 2-dimensional
3D: 3-dimensional
3DCG: 3-dimensional computer graphics
HSUM: Health Sciences University of Mongolia
LMS: learning management system
