# Supplementary material for: The Effectiveness of an Interactive 3-Dimensional Computer Graphics Model for Medical Education
Source: Interact J Med Res. 2012 Jul 9;1(2):e2. doi: 10.2196/ijmr.2172 (PMC3626131; doi:10.2196/ijmr.2172)
Supplement: Supplementary file 1 [file ijmr_v1i2e2_app1.pptx]

## Slide 1
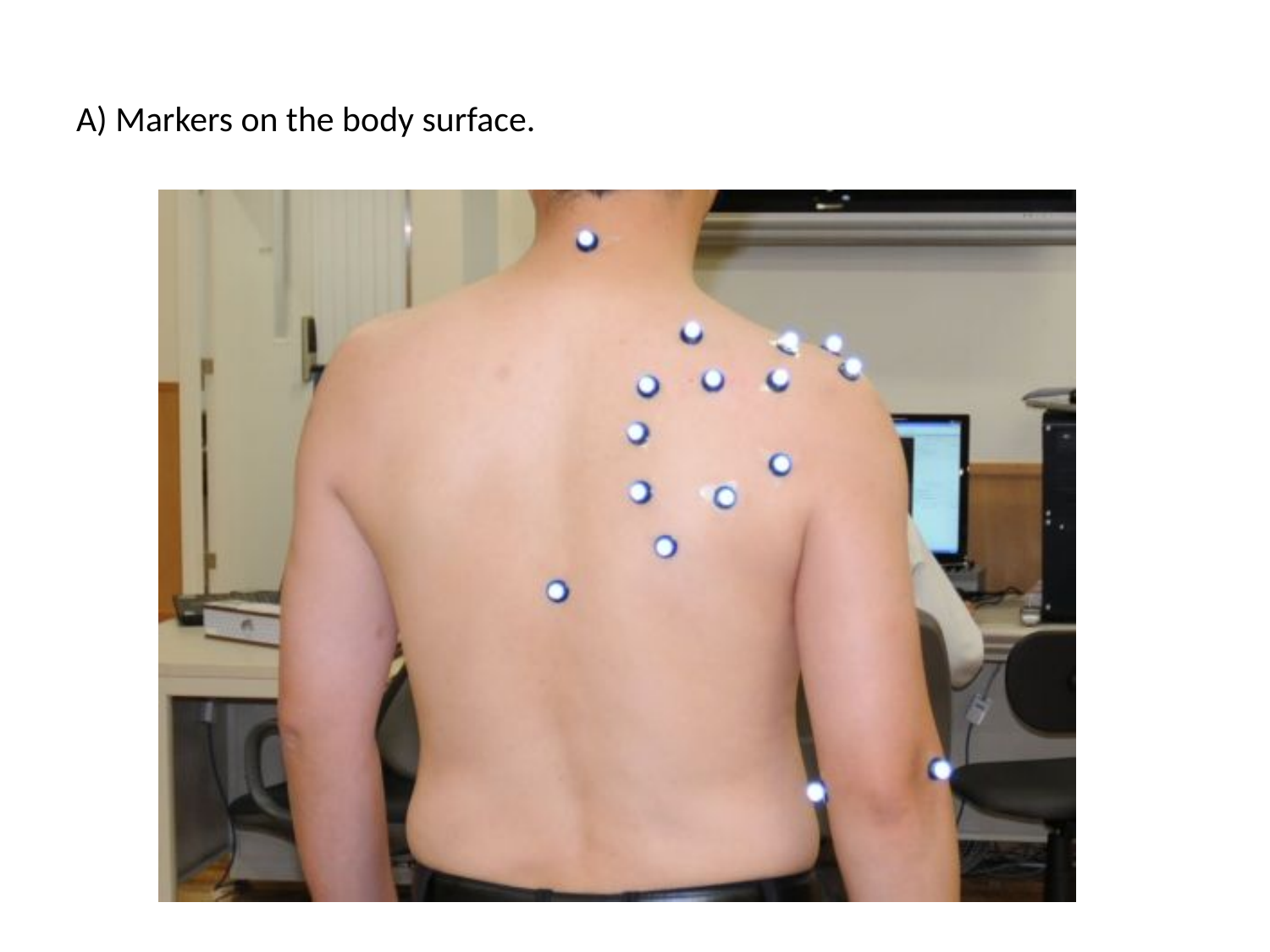

# A) Markers on the body surface.

## Slide 2
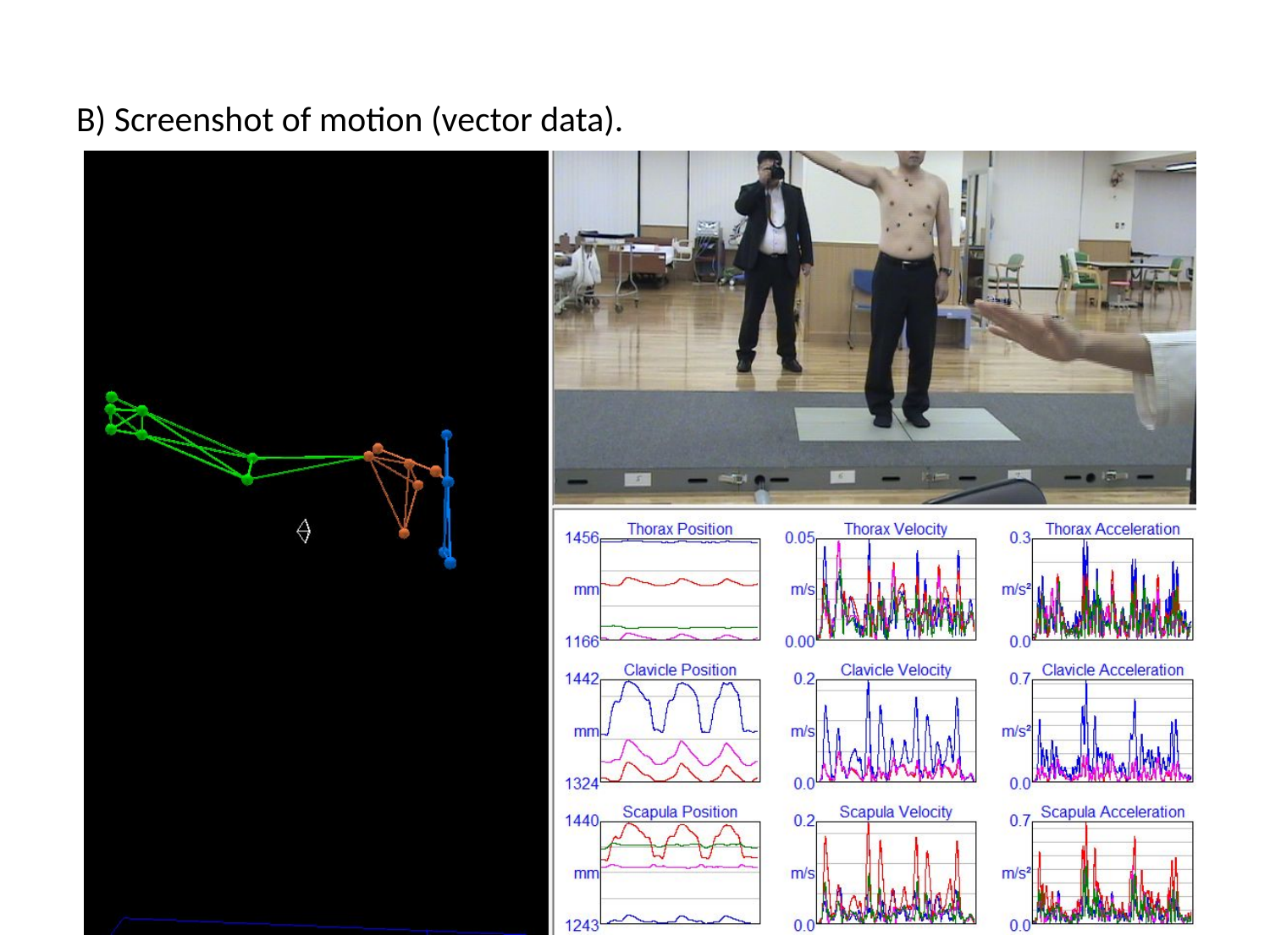

# B) Screenshot of motion (vector data).

## Slide 3
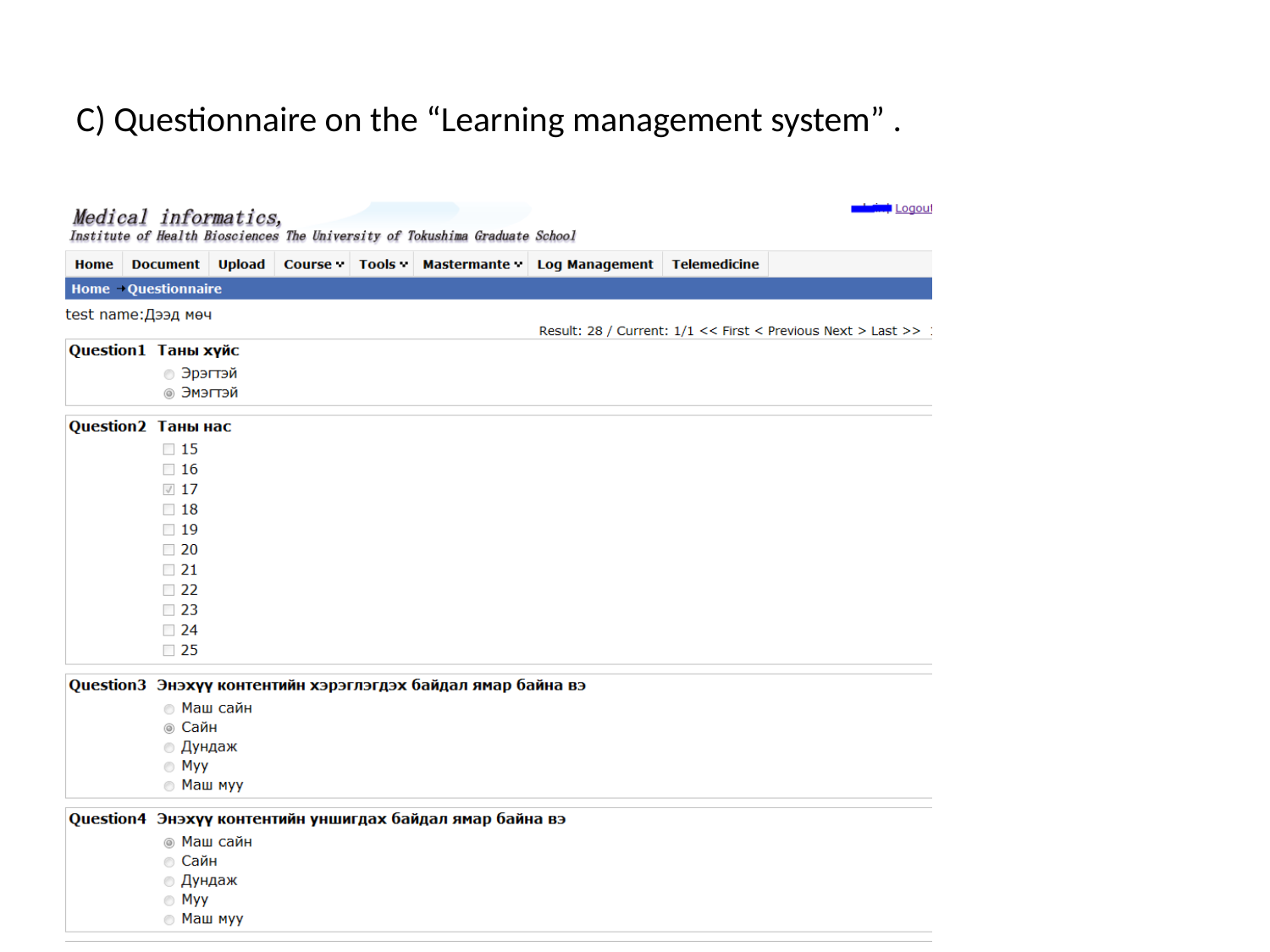

# C) Questionnaire on the “Learning management system” .
